# Strategies for prostate cancer prevention: Review of the literature

**DOI:** 10.4103/0970-1591.42608

**Published:** 2008

**Authors:** H. Krishna Moorthy, P. Venugopal

**Affiliations:** Division of Urology, Lakshmi Hospital, Kochi, and Department of Surgery, Mangalore; 1 Sr.Consultant in Urology, Mangalore

**Keywords:** Chemoprevention, prostate cancer

## Abstract

The goal of primary chemoprevention is to decrease the incidence of a given cancer, simultaneously reducing treatment-related adverse events, cost of treatment of the disease and mortality. Prostate cancer is an attractive and appropriate target for primary prevention because of its high incidence and prevalence, increased disease-related mortality, long latency and molecular pathogenesis and epidemiological data indicating that modifiable environmental factors may decrease risk. Various agents have been suggested to prevent prostate cancer and many clinical trials are currently on. Ultimately the adoption of a preventive strategy hinges on its potential benefits weighed against the potential risks of the specific agents used. This article is aimed to examine the experimental and epidemiological data spanning a period of 1998 to 2007, demonstrating the chemopreventive activity, safety and toxicity of various nutritional elements and other agents that can help prevent prostate cancer and/or slow disease progression.

## INTRODUCTION

Prostate cancer is a slow-growing tumor of older men, constituting the most common type of non-skin cancer and the second leading cause of cancer-related deaths in American men. In the late 80s and early 90s great attention was given to screening asymptomatic men by measuring concentration of prostate specific antigen (PSA), which eventually led to a significant increase in the detection of clinically insignificant tumors. Despite this increase, mortality due to prostate cancer has decreased every year since 1992. Though the exact pathogenesis is not clear, epidemiological evidence supports a relationship between prostate cancer and serum levels of testosterone.[1] Other risk factors include advanced age, family history, African-American ethnicity, poor diet and cadmium exposure.[2] The frequency of prostate cancer increases exponentially with advanced age and the natural progression to prostate cancer tends to be more aggressive in younger men and those with a family history of the disease.

Although controversial, strategies for decreasing prostate cancer mortality have focused on early detection. The most important tumor marker for detection and post-treatment monitoring of prostate cancer is PSA level. However, PSA levels have fallacies. Increased levels of serum PSA can occur in prostate cancer, benign prostatic hyperplasia and prostatitis. Conversely, treatment with 5 alpha reductase inhibitors lowers PSA levels to approximately 50%. However, PSA assessment still remains the best method to detect cancers at a pre-symptomatic stage. Newer methods for increasing the sensitivity and specificity of PSA screening tool are currently being adopted.

## MATERIALS AND METHODS

To identify the various strategies for prostate cancer chemoprevention, a MEDLINE search (from 1970 to 2007) and bibliographic search of the English language literature was conducted.

## CHEMOPREVENTION

Carcinogenesis is a multi-step molecular process induced by genetic and epigenetic changes that disrupt the balance between cell proliferation, apoptosis, differentiation, senescence and the pathways controlling these cellular processes. Precursor lesions that represent intermediate stages between normal and malignant cells can arise as long as 20 years before the appearance of cancer. Chemoprevention is defined as the use of natural or synthetic agents that reverse, inhibit or prevent the development of cancer in cancer-free individuals.[3] Important to chemoprevention is the fact that carcinogenesis is a process over time involving cellular growth and division and hence inhibition or slowing this process can potentially prevent cancers from becoming clinically significant.[4] This approach relies on targeting healthy persons at risk of developing the cancer in question and using agents with a low probability of inducing adverse events, similar to the use of Tamoxifen for prevention of breast cancer in high-risk women.[5] The high rate of occurrence and long lead time to development of clinically significant prostate cancer make this disease ideal for study of chemoprevention,[6,7] though this long lag period may not be exactly ideal for assessing the results of various scientific studies. Prostate carcinogenesis includes progression from normal appearing epithelium to dysplasia (low-grade prostatic intraepithelial neoplasia or LGPIN) to severe dysplasia (high-grade prostatic intraepithelial neoplasia or HGPIN) and finally to invasive prostate cancer. Various agents are considered for preventive treatment of prostate cancer which can either be used in primary prevention which includes the period prior to the diagnosis of prostate cancer or in secondary prevention which may involve the prevention of recurrence or progression of micrometastatic disease.

## NUTRIENTS AND VITAMINS

Evidence from epidemiological studies suggests associations between exposure to a variety of dietary nutrients and certain cancers including lung, colon, breast and prostate.[8] The role of dietary constituents and vitamins in the chemoprevention of prostate cancer has been addressed in several clinical trials. Most of the available data consist of epidemiologic or retrospective studies and should be interpreted with attention to potential confounders.[9]

### Carotenoids

There are a number of carotenoids with antioxidative activity. Lycopene is a red-orange, highly unsaturated, acyclic isomer of beta-carotene found primarily in tomatoes and tomato-derived products and in other red fruits and vegetables. Lycopene possesses significant antioxidant properties that may confer some antineoplastic activity. Studies have found out that increased lycopene intake was associated with a decreased risk of prostate cancer.[10–12] However, another study[13] did not show a reduced risk of prostate cancer with beta-carotene supplementation. Another interesting study is that intake of vitamin A from plant sources was associated with decreased prostate cancer risk while the intake of vitamin A from animal sources was associated with increased risk.[14] This may be due to the higher fat content in the diet of men with high animal vitamin A intake. An interesting study by Giovannucci et al.,[15] has shown that eating at least two servings per week of tomato sauce can significantly decrease the risk of developing prostate cancer. Tomato paste and other processed tomato products are even more effective than fresh tomatoes in preventing prostate cancer[16] since tomato processing actually increases the bioavailability of lycopene to humans. This is because processing converts much of the trans-form of lycopene found in fresh tomatoes into the cis-form which is much readily taken up in humans.

### Dietary fat

Several studies support a positive correlation between some aspect or component of animal fat and prostate cancer risk.[17,18] Populations with higher fat intake in diet have increased prostate cancer relative risks by a factor of 1.6-1.9.[19] High dietary fat may have multi-factorial role in the causation of prostate cancer. Though it is established that a high-fat diet can increase serum androgen levels, the precise mechanism for increased production of sexual hormones is not clearly understood. Plasma concentrations of fatty acids are increased with increasing consumption of fat and these plasma fatty acids inhibit binding of gonadal steroids to sex hormone binding globulins.[20] Thus a high fatty diet may increase prostate cancer risk by causing long-term androgenic stimulation. A low-fat, high-fiber diet increases fecal excretion of gonadal hormones and possibly lowers serum androgen levels and thereby lowers incidence of prostate cancer.[21]

Consumption of oily fish and other foods rich in omega-3 fatty acids may help prevent the spread of carcinoma prostate.[22] The omega-3 fatty acids interfere with functions of omega-6, which cancer cells may use as a source of energy and prevent them from spreading beyond the prostate.

### Soy

The diet in many Asian countries is especially high in plant products including soy.[23] Higher consumption of soy milk has been found to lower the risk of prostate cancer by 70%.[24] Southeast Asian men consume up to 50 times more soy daily than their Western counterparts and demonstrate a 10-fold lower incidence of prostate cancer and prostate cancer mortality. Several natural anti-carcinogens (such as protease inhibitors, phytates, phytosterols, saponins, lignans and isofavones) have been identified in soybeans.[25,26] After structural modifications by intestinal bacteria, isoflavones are converted to compounds that possess weak estrogenic and anti-estrogenic properties. Phytoestrogens found in soy products increase serum sex hormone binding globulin via increased hepatic synthesis which then decreases bioavailability of testosterone. Isofavonoids may also prevent prostate cancer by weakly binding androgen hormone receptors in the prostate thereby interfering with androgenic stimulation of prostate cells.[27] Genistein, an isoflavone has also received much attention due to its interesting antiproliferative, estrogenic and antiestrogenic effects in prostate cancer cells.[28,29]

### Green tea

Epidemiological studies have shown that there is a low incidence of prostate cancer among native Asian men with a high dietary intake of green tea and hence the polyphenols contained in green tea have been proposed as chemopreventive.[30] Prostate cancer cell culture and gene expression experiments have demonstrated that the major polyphenolic constituent of green tea inhibits cell growth and dysregulates the cell cycle.[31]

### Vitamin E

α-tocopherol is the most prevalent chemical form of vitamin E found in vegetable oils, seeds, grains, nuts and other foods. It is a potent antioxidant and has been suggested as a potential preventative of several cancers, particularly lung cancer.[32] In the Alpha Tocopherol, Beta Carotene Cancer Prevention Study,[33] it has been suggested that α-tocopherol administration reduces prostate cancer risk by reducing the incidence of prostate cancer and even reducing prostate cancer mortality. Many clinicians have been in the habit of advising higher doses of vitamin E, often 400 IU/day; however, recently published evidence suggests that the recommended dose should be < 150 IU/day.[34] Some other studies suggest an inverse relation between blood α-tocopherol levels and prostate cancer risk.[35] Vitamin E has been theorized to prevent progression of latent tumors to more invasive disease.[36] The antioxidant property of vitamin E prevents the propagation of free radical damage in biologic membranes and to critical cellular structures like DNAs and proteins. Vitamin E may also protect by enhancing immune function and lowering the activity of protein kinase C, a cellular signal transducer that regulates cell proliferation.

### Selenium

Selenium found in many vegetables and grains grown in selenium-rich soil has antioxidant activity and has other direct effects on tumor cells.[23] Higher level of serum selenium has been found to inhibit the growth of prostate tumor cells in vitro.[37] One of the randomized placebo-controlled trials done on skin cancer revealed a statistically significant reduction of prostate cancer incidence of 63% when 200 microgram per day of selenized yeast was given.[38] The protective effect of selenium appeared to be most prominent in men with low baselines levels of PSA (< 4 ng/ml) and low baseline plasma selenium concentrations.

The Selenium and Vitamin E Cancer Prevention Trial (SELECT) was started to evaluate the role of vitamin E and selenium supplementation in preventing prostate cancer.[39] This prospective, randomized, double-blind, placebo-controlled prevention trial involved healthy men with a normal digital rectal examination and a serum PSA level below 4 ng/ml. Subjects were randomized to one of four treatment groups: vitamin E (400 mg racemic α-tocopherol) plus selenium (200-μgram 1-selenomethionine), vitamin E plus placebo, selenium plus placebo or placebo plus placebo. A minimum follow-up period of seven years was planned. Enrollment of patients began in August 2001 and was closed in June 2004 with 35,534 participants: final study results are anticipated in 2013. Unlike trials with selenium and vitamin E reported so far, in which prostate cancer was a secondary end point, clinical diagnosis of prostate cancer is the primary end point of SELECT.

### Vitamin D

Variation in the degree of exposure to sunlight affects the blood levels of vitamin D and calcium in the population. Studies have shown that men with higher levels of serum calcium have a four to fivefold elevated risk of metastatic prostate cancer.[11,40] The active metabolite of vitamin D, namely calcitriol inhibits growth of both primary cultures of human prostate cancer cells and cancer cell lines by a mechanism not clearly understood. Calcitriol may affect cell cycle progression and also initiate apoptosis. The current recommended dose of vitamin D is 10 μgram/day.[41] Since overdoses of vitamin D can result in hypercalcemia, analogues of calcitriol with lesser side-effects, but having more potent anti-proliferative effect have been developed.[42]

Summary: Food rich in lycopenes (like tomatoes especially as processed tomatoes), omega 3 fatty acids (fish oils), isoflavonoids (soybeans), polyphenols (green tea), α-tocopherol (vegetable oils, seeds, grains, nuts), selenium (vegetables, grains), vitamin D and fiber can significantly reduce the risk of developing prostate cancer or even reduce the mortality due to the disease.

## NON-STEROIDAL ANTI-INFLAMMATORY DRUGS

Non-steroidal anti-inflammatory drugs act to prevent the synthesis of endogenous prostaglandins by inhibition of the cyclo-oxygenase (COX) enzyme. Expression of the COX-2 isoenzyme is three to five times greater in patients with prostate cancer than in those with benign prostatic hyperplasia and is responsible for increased angiogenesis, tumorigenesis and prostate cancer growth.[43,44] Non-steroidal anti-inflammatory drugs (NSAIDs) such as aspirin, sulindac and ibuprofen are reported to have prostate cancer chemopreventive activity; regular intake of NSAIDs actually reducing the prostate cancer risk.[45] A meta-analysis of the combined results from 12 studies of NSAIDs and prostate cancer risk found a 10% risk reduction associated with aspirin therapy,[46] although the risk associated with other NSAIDs was more variable. However, with the recent evidence regarding increased cardiovascular risk with selective COX-2 inhibitors, it is unclear whether continuous administration of these agents can be justified.

Summary: Non-steroidal anti-inflammatory drugs (NSAIDs) such as aspirin, sulindac and ibuprofen are reported to have prostate cancer chemopreventive activity.

## SELECTIVE ESTROGEN-RECEPTOR MODULATORS (SERMs)

Interest in SERMs as preventive agents is stimulated by an apparent role of estrogens in the pathogenesis of prostate cancer through promotion of cell growth.[47] Furthermore, age-related prostate disease rates parallel increases in serum estrogen levels and there is a low incidence of prostate cancer in cultures with diets rich in phytoestrogens.[28]

Summary: Phytoestrogens and SERMs are found to have prostate cancer prevention potential in experimental studies.

## 5-ALPHA-REDUCTASE INHIBITORS

It is well known that dihydrotestosterone is the principal androgen responsible for normal and hyperplastic growth of prostate and is 10 times more potent than testosterone. 5-alpha-reductase inhibitors prevent the conversion of testosterone to dihydrotestosterone. Of the two isoenzymes of 5-alpha-reductase, Type 2 is present in normal and hypertrophic prostate tissue, whereas Type 1 is the predominant form in prostate cancer cells and is over-expressed in some prostate cancers. Because androgens are required for the development of prostate cancer[48] and men with lower 5-alpha-reductase activity have lower rate of prostate cancer, 5-alpha-reductase inhibitors have been considered for chemo-prevention of prostate cancer. 5-alpha-reductase inhibitors decrease the prostate levels of dihydrotestosterone and reduce the androgenic stimulation to the prostate, although the systemic androgenic effects are only mildly affected. *In vitro* studies also showed that the growth of previously established prostate cancer lines were inhibited by 5-alpha-reductase inhibition.[49,50] Out of the two common types of 5-alpha-reductase inhibitors available, finasteride selectively inhibits the Type 2 isoenzyme of 5-alpha-reductase, whereas dutasteride inhibits both izoenzymes. The effect of finasteride as a chemopreventive agent has been established in the critical analysis of the Prostate Cancer Prevention Trial (PCPT) study by Goetzl and Holzbeierlein.[51] In the PCPT study, healthy men aged 55 years or older with a normal digital rectal examination and a PSA level of 3 ng/ml or lower were enrolled and a total of 18,882 men were randomized to receive either finasteride 5 mg/day or placebo for seven years. Subjects were monitored annually with a digital rectal examination and PSA level measurement. For those receiving finasteride, total PSA level was adjusted for the effect of finasteride before being reported. The prevalence of prostate cancer was reduced by 24.8% in the finasteride arm compared to the placebo arm. However, there was a relative increase in the frequency of invasive tumors in the finasteride group - one explanation being that finasteride treatment effect not only selectively inhibits low-grade tumors, but also promotes high-grade tumors. Ultimately, the adoption of a preventive strategy always hinges on its potential benefits weighed against the potential risks and recommendations for use of finasteride for prostate cancer prevention.[30] The potential benefits of finasteride when used to prevent prostate cancer include the reduction in cancer prevalence, fewer urinary symptoms and lower risk of acute urinary retention, while the drawbacks include the 1.3% increase in high-grade tumors requiring more aggressive therapy and having potential excess mortality, sex-related adverse effects and the cost of treatment of such conditions and cost of finasteride.

A Phase II, double-blind, placebo-controlled, dose-ranging comparative trial clearly demonstrated that serum dihydrotestosterone suppression was significantly greater with the dual (Type 1 and 2) 5-alpha-reductase inhibitor dutasteride 0.5 mg/day than with finasteride 5 mg/day in men with benign prostatic hyperplasia.[52] The Reduction by Dutasteride of Prostate Cancer Events (REDUCE) trial[53] was aimed to study the effect of dutasteride 0.5 mg/day in men with an increased risk of developing prostate cancer. Free and total PSA levels were assessed every six months throughout the study and prostate biopsy performed at two and four years. Study enrollment was completed in 2005 and the results are awaited.

However, other androgen blockers like flutamide, bicalutamide and nilutamide are associated with too many side-effects to be of practical use in the asymptomatic healthy population, unlike finasteride.[54]

Summary: 5-alpha-reductase inhibitors such as finasteride and dutasteride have been recommended as chemotherapeutic agents for prevention of prostate cancer.

## OTHER POSSIBLE AGENTS

Studies by Giovannucci and Clinton[55] have shown there was no clear association between dietary factors like vitamin C, vitamin B_1_, vitamin B_2_, niacin, zinc, protein and carbohydrates with prostate cancer. However, other drugs like nonclassic antioxidant agents including the polyphenols, the isothiocyanates, difluromethylornithine, oltipraz and N-acetyl cysteine have been proposed as potential chemopreventive agents.[56,57]

Many chemotherapeutic agents including Silibinin, Inositol hexaphosphate, Hecursin, Apigenin, Acacetin and Epigallocatechin-3 gallate have been identified in laboratory studies to be of use in the management of prostate cancers.[58] The extract of pomegranate from the tree Punica granatum possesses strong antioxidant and anti-inflammatory properties and has been found to inhibit human prostate cancer cell growth in *in vitro* and *in vivo* preclinical models.[59] Similarly grape seed extract has been found to inhibit growth and induce apoptotic death of human prostate cancer cells in culture.[60] Grape seed extract causes caspase 3 and caspase 9-mediated apoptosis.

Phytochemicals are nonnutritive components of plants that are currently being studied in chemoprevention of various diseases for their pleiotropic effects and non-toxicity. Various studies[61–63] have shown the therapeutic potential of curcumin, which is the active constituent of turmeric widely used as a spice in Indian cooking, in human prostate cancer. Curcumin has anti-inflammatory, antioxidant and anti-tumor properties.[64] The mechanisms proposed are that curcumin induces apoptosis in both androgen-dependent and androgen-independent prostate cancer cells, inhibits proliferation and angiogenesis of LNCaP prostate cancer cells and inhibits tyrosine kinase activity of epidermal growth factor receptor and depletes the protein.

Sanguinarine, an alkaloid derived from the bloodroot plant *Sanguinaria canadensis* has been shown to possess anti-microbial, anti-inflammatory and antioxidant properties. It has been shown that sanguinarine can cause cell cycle blockade and apoptosis of human prostate carcinoma cells via modulation of cyclin kinase inhibitor-cyclin-cyclin dependent kinase machinery.[65] The growth inhibitory and antiproliferative effects of sanguinarine in human carcinoma cells was irrespective of their androgen status.

Encouragingly, it was recently proposed that the consumption of red wine might be protective against prostate cancer.[66] Every additional glass of red wine drunk per week showed statistically significant 6% decrease in relative risk of prostate cancer and men drinking four to seven glasses per week were almost 25% less likely to have the disease (a relative risk reduction of 48%). Resveratrol is the candidate agent in red wine that is thought to protect against cancer. Resveratrol is a naturally occurring plant antibiotic found in grape skins and red wine and has antioxidant activity, anti-platelet aggregation effect, anti-atherogenic property, estrogen-like growth promoting effect, growth-inhibiting activity, immunomodulation and chemoprevention of cancer. Resveratrol metabolizes into the anti-leukemic agent piceatannol, which may provide a novel explanation for the cancer-preventive properties of resveratrol. However, other alcoholic beverages did not have similar protective effects.

Eating at least five servings a week of cruciferous vegetables such as broccoli and cauliflower can significantly decrease the risk of developing prostate cancer.[67] Sulforaphane is the compound having chemopreventive role in these vegetables.

Zyflamend, a unique herbal preparation with non-selective COX-inhibitory activity, induces apoptosis of prostate cancer cells that lack COX-2 expression.[68] Zyflamend is composed of 10 potency assured herbal extracts: rosemary (*Rosmarinus officianalis*), turmeric (*Curcuma longa*), ginger (*Zingiber officinale*), basil (*Ocimum basilicum*), green tea (*Camellia sinensis*), Japanese knotweed (*Polygonum cuspidatum*), Chinese goldthread (*Soptis* spp), barberry (*Berberis* spp), Oregano (*Origanum vulgare*) and Chinese or Baikal skullcap (*Scutellaria baicalensis*).

Summary: Nonclassic antioxidant agents like polyphenols, isothiocyanates, difluromethylornithine, oltipraz and N-acetyl cysteine and chemotherapeutic agents including Silibinin, Inositol hexaphosphate, Hecursin, Apigenin, Acacetin and Epigallocatechin-3 gallate have all been proposed to have antineoplastic properties. The extracts of pomegranate and grape seed, curcumin, sanguinarine, an alkaloid derived from the bloodroot plant Sanguinaria canadensis, resveratrol in red wine and sulforaphane in cruciferous vegetables can also significantly decrease the risk of developing prostate cancer.

## THERAPEUTIC VACCINES FOR PROSTATE CANCER

Vaccines for prostate cancer, which for several years have been shown to generate immunological responses, are beginning to show significant clinical promise.[69] Dendritic cell vaccines (DCs) are highly proficient antigen-presenting cells that localize to multiple epithelial sites. The addition of proteins like prostate-specific membrane antigen (PSMA-DCVax-Prostate vaccine) or prostatic acid phosphatase GM-CSF fusion protein (Provenge) to such vaccines appears to have beneficial effects in prolonging survival in carcinoma prostate. Whole-tumor cell vaccines have been investigated for some years. The GM-CSF secreting vaccine GVAX is an admix of the prostate cancer cell lines PC-3 and LNCaP whereas Onyvax-P consists of irradiated cell lines from Ony-Cap 23, LNCaP and P4E6. Both these vaccines are currently undergoing Phase III studies following evidence that they reduce PSA velocities, extend time to progression and are well tolerated. Pox virus-based vaccines utilizing live recombinant vaccinia (PROSTVAC) or recombinant fowlpox, induce expression of tumor-associated antigens and stimulate T cell activation. However, the efficacy and long-term safety of these vaccines are currently being assessed in various trials.

Summary: Trials on vaccines offer great promise for prevention of prostate cancer in the near future.

## EPILOGUE

Identifying pharmacologic and nutritional preventive strategies for prostate cancer remains a challenge for physicians and researchers. Though prevention of prostate cancer seems to be novel approach, it is yet to be understood who would benefit from these drugs. It is logical to understand that people with genetic predisposition are less likely to benefit than men at risk for sporadic cancer. The efficacy of the drugs will also have to be proved by large randomized clinical trials of long duration. Biomarkers of carcinogenesis, tailored to the agent under investigation are essential in the clinical development of chemopreventive agents. Prostatic intraepithelial neoplasia (PIN) is an intraluminal proliferation of secretory cells of the prostate duct-acinar system.[70] Common genetic alterations in PIN and prostate cancer have been identified: gain of Chromosome 7, loss of 8p, gain of 8q and loss of 10q, 16q and 18q.[71] While low-grade PIN has unclear predictive value for malignancy, high-grade PIN is suspected to be the precursor to prostate carcinoma because of the similarities in histological diagnosis. Recent speculation suggests that other histological changes such as metaplasia and atrophy may also be important precursors for prostate cancer. However, it is not clear whether chemopreventive strategies could be decided by an invasive test like prostate biopsy, which can pick up these lesions.

Androgen receptor (AR) plays a key role as a transcriptional factor in prostate development and carcinogenesis. Identification of androgen-regulated genes is essential to elucidate the AR pathophysiology in prostate cancer. By combining chromatin immunoprecipitation (ChIP) with tiling microarrays (ChIP-chips), the androgen target genes that are directly regulated by AR in LNCaP cells have been identified.[72] This will enable us to extend our knowledge of the diversity of the androgen genetic network and steroid action in prostate cancer cells. Better understanding of the molecular biology of prostate cancer is also essential to identify the best chemopreventive agent. Another controversy would be the endpoint of these studies, whether it is the prevention of death, regression of intraepithelial neoplasia or the decline in PSA both in men with intact prostate and in men with PSA relapse after radical prostatectomy.[22] One interesting study by Uzzo *et al*.,[73] showed that a high proportion of men at risk for prostate cancer self-initiated nutritional therapies in the form of various nutritional, vitamin and mineral supplements. Leiberman[7] has suggested adopting a public health approach for prevention of prostate cancer including 1. changes in dietary practices - increased fruits and vegetables and decreased carbohydrates and charbroiled meat, 2. caloric restriction/obesity control with reduction in serum IGF-1, 3. increased physical activity and stress reduction and 4. early detection of precancerous lesions such as PIA (proliferative inflammatory atrophy, which is now considered as the earliest precursor lesion for prostate cancer), IEN (intraepithelial neoplasia) and early cancer. In addition, a cost-effective pharmacologic medically oriented translational science strategy is being developed. This includes 1. identification of individuals at risk using clinical, histological, genetic and proteomic profiles, 2. risk reduction by chemopreventive agents through modulation of surrogate endpoints such as IEN, 3. suppression/reversal of promoter CpG methylation in target genes-GSTP1, RAR-beta etc, 4. suppression/reversal of signature protein patterns of early prostate cancer with delay in the onset of clinically active prostate cancer. The recent concept of replication-competent adenovirus-mediated suicide gene therapy in which an oncolytic adenovirus armed with chemoradiosensitizing genes is combined with intensity-modulated radiotherapy (IMRT) and used to destroy tumor cells experimentally[74] provides a beacon of hope to provide a potential long-term benefit to patients with carcinoma of prostate.

For the common man, the bottom line of prostate cancer prevention would be to consume a variety of fruits and vegetables, reduce the intake of white bread, meats and saturated fat, reduce or eliminate smoking, reduce stress, sleep well, exercise, avoid unnecessary hormone use, particularly androgens, eat more fiber and fish oil. However, it is worthwhile for the treating physician to understand the various agents useful in primary prevention which includes the period prior to the diagnosis of prostate cancer and in secondary prevention which may involve the prevention of recurrence or progression of micrometastatic disease. An ounce of prevention is worth a pound of cure.
